# CD11c^+^ MHCII^lo^ GM-CSF-bone marrow-derived dendritic cells act as antigen donor cells and as antigen presenting cells in neoepitope-elicited tumor immunity against a mouse fibrosarcoma

**DOI:** 10.1007/s00262-018-2202-4

**Published:** 2018-07-20

**Authors:** Hakimeh Ebrahimi-Nik, William L. Corwin, Tatiana Shcheglova, Alok Das Mohapatra, Ion I. Mandoiu, Pramod K. Srivastava

**Affiliations:** 1 Department of Immunology, School of Medicine, Carole and Ray Neag Comprehensive Cancer Center, University of Connecticut, 263 Farmington Ave, Farmington, CT 06030-1601 USA; 2 Department of Computer Science and Engineering, University of Connecticut, Storrs, CT USA

**Keywords:** Cancer, Immunotherapy, Neoepitopes, Vaccine, Dendritic cells, Adjuvant

## Abstract

Dendritic cells play a critical role in initiating T-cell responses. In spite of this recognition, they have not been used widely as adjuvants, nor is the mechanism of their adjuvanticity fully understood. Here, using a mutated neoepitope of a mouse fibrosarcoma as the antigen, and tumor rejection as the end point, we show that dendritic cells but not macrophages possess superior adjuvanticity. Several types of dendritic cells, such as bone marrow-derived dendritic cells (GM-CSF cultured or FLT3-ligand induced) or monocyte-derived ones, are powerful adjuvants, although GM-CSF-cultured cells show the highest activity. Among these, the CD11c^+^ MHCII^lo^ sub-set, distinguishable by a distinct transcriptional profile including a higher expression of heat shock protein receptors CD91 and LOX1, mannose receptors and TLRs, is significantly superior to the CD11c^+^ MHCII^hi^ sub-set. Finally, dendritic cells exert their adjuvanticity by acting as both antigen donor cells (i.e., antigen reservoirs) as well as antigen presenting cells.

**Electronic supplementary material:**

The online version of this article (10.1007/s00262-018-2202-4) contains supplementary material, which is available to authorized users.

## Introduction

Dendritic cells (DCs) are professional antigen presenting cells (APCs), which play a critical role in initiating and modulating immune responses [1–4]. DCs show considerable heterogeneity in terms of functions, lineages and phenotypes [5]. In addition to their roles in vivo, DCs have been employed as natural adjuvants in a variety of tumor vaccines albeit with very limited success. Out of practical necessity, such studies have mostly utilized bone marrow-derived DCs (BMDCs) in mice [6–8] and monocytic-derived DCs in humans, even though these particular DC populations have little physiological relevance in vivo. Sipuleucel-T, the first DC-based vaccine approved by United States Food and Drug Administration, was aimed at treatment of a sub-set of patients with prostate cancer. Although approved on basis of randomized clinical trials, this DC-based vaccine had extremely limited clinical activity [9, 10].

Despite this poor track record, the distinct ability of DCs to uptake and process antigens and prime naïve CD8^+^ T cells in vivo [11], exerts a powerful pull on tumor immunotherapy. A large number of studies have indeed probed the adjuvanticity of DCs [12–21]. Therefore, mechanistic studies on DC-based vaccines remain of great foundational and translational interest. Such studies, pursued mostly using the chicken egg ovalbumin, a well-characterized model xeno-antigen in mouse models of tumor immunity, which has led to diametrically opposed interpretations of the mechanism by which a DC-based immunogen works. Livingston and Kuhn [22] showed that upon immunization with SIINFEKL-pulsed DCs, the immunizing DCs could directly prime the host CD8^+^ T cells without the participation of endogenous APCs. The immunizing DC thus acted as the APC. On the other hand, Yewdall et al. [23] observed that BMDCs were not able to directly prime CD8^+^ T cells and could only act as antigen reservoirs, i.e., they acted as antigen donor cells (ADC) and not APC. This mechanistic distinction, and its resolution are important for understanding of how exogenous DC behave in vivo, and also for translational purposes in cancer immunotherapy.

Towards that goal, and in the current context of great excitement for neoepitopes of cancers [24–26], we have tested the efficacy and mechanism of DC-based vaccination in tumor rejection using a mutated neoepitope of a chemically induced fibrosarcoma, instead of a model antigen such as ovalbumin. Here, testing a variety of APCs including DC sub-sets, we conclude that a GM-CSF-CD11c^+^ MHC class II ^low^ DC sub-set has the most powerful adjuvanticity of all the tested APCs and that these APCs act as both an ADC as well as an APC in vivo.

## Materials and methods

### Mice, tumors and peptides

C57BL/6J, BALB/cJ, C57BL/6-Tg (TcraTcrb) 1100Mjb/J (OT-I Tg mice) and B6.129P2-B2mtm1Unc mice (6-week female) were purchased from the Jackson Laboratory, and maintained in our virus-free mouse facilities under approval from the Institutional Animal Care and Use Committee. CD45.1^+^ RAG^−/−^ OT-I TCR Tg mice (OT-I) were bred and housed in barrier facilities maintained by the Center for Laboratory Animal Care (CLAC). These mice have transgenic T-cell receptor that is designed to recognize the complex of H2Kb and ovalbumin peptide residues 257–264. Meth A cells that have been in our lab since 1988, were originally obtained from Lloyd J Old. Meth A ascites cells were used for passage. B16 melanoma cells that were permanently transfected with ovalbumin antigen (B16-OVA) were generously gifted by Dr. Nick Restifo (Center for Cancer Research, National Cancer Institute, Bethesda, MD, USA). Neo1, a neoepitope of the BALB/cJ Meth A fibrosarcoma, was synthesized by JPT Peptide Technologies GmbH.

### Immunization

Splenocytes, day 7 granulocyte-macrophage colony-stimulating factor-derived BMDCs, (GM-CSF-BMDCs), day 10 FMS-like tyrosine kinase 3 ligand BMDCs (FLT3L-BMDCs), bone marrow-derived macrophages (BMDMs) and day 3 monocyte-derived DCs (Mo-DCs) were pulsed with 40 µg Neo1 (1 µl of a 20 mM Neo1 peptide solution was added to 7.5 million BMDM, BMDC or 500,000 MO-DCs in 200 µl RPMI medium for final peptide concentration of 100 µM) or chicken ovalbumin-derived long peptide 18-mer (LP) LEQLKSIINFEHLKEWTS (referred to a LP SIINFEHL) for 2 h. The BMDM, BMDC or MO-DC cultures were washed, and used to immunize a single mouse. (The actual quantity of peptide that is actually loaded into the DCs, and used to immunize each mouse, is thus unknown.) The LP contains the dominant K^b^-restricted epitope SIINFEHL within it. The natural epitope is SIINFEKL; however, SIINFEHL has been shown to be equivalent with respect to its interaction with the T-cell receptors [27, 28]. The LP, as opposed to the precise peptide, is used because it gives more consistent results [29]. All immunizations were carried out in presence of CTLA4 blockade, using the IgG2b isotype (Clone: 9D9, Bio X Cell), administered with the second immunization and every 3 days after tumor challenge. In unpublished studies, we have demonstrated that certain neoepitopes demonstrate their fullest activity only in combination with CTLA4 blockade.

### T cells depletion

BALB/cJ mice were injected with 250 µg of ISO, CD8 (Rat IgG2b, clone 2.43, Bio X Cell) or CD4 (Rat IgG2b, clone GK1.5, Bio X Cell) depletion antibodies 2 days before each immunization and tumor challenge and every week afterwards.

### BMDCs and BMDMs

Bone marrow cells (2–3 million per 10 cm^2^ bacteriological Petri dishes) of 6- to 8-week-old mice were cultured in complete RPMI supplemented with 20 ng per ml recombinant murine GM-CSF (Peprotech) and incubated at 37 °C for 7 days to generate GM-CSF-BMDCs. Bone marrow cells (10 million per 10 cm^2^ bacteriological Petri dishes) of 6- to 8-week-old mice were cultured in complete RPMI supplemented with 200 ng per ml recombinant murine FLT3L (TONBO biosciences) and incubated at 37 °C for 10 days to generate FLT3L-BMDCs.

BMDMs were generated by flushing femurs and tibias with DMEM. Cells were incubated overnight in 25 cm^2^-flasks at 37 °C. The following day, 10^7^ of the suspended cells were harvested and maintained in 10 cm^2^ bacteriological Petri dishes (BD-Falcon) with DMEM supplemented with 10% FBS, 20% L929-cell conditioned media [supernatant of L929 cells which contains Macrophage colony-stimulating factor (M-CSF)] and supplements. Cultures were fed with 5 ml of the medium after 3 days. 7 days after the isolation, cell monolayers (BMDMs) were exposed to ice-cold PBS and recovered by scraping.

### Mo-DCs

Peripheral blood mononuclear cells (PBMCs) were isolated using lymphoprep and SepMate (StemCell Technologies). Monocytes were isolated from PBMCs using EasySep™ Mouse Monocyte Isolation Kit. 500,000 monocytes/ml were cultured in complete RPMI with FBS supplemented with 50 ng/ml GM-CSF (Peprotech) plus 25 ng/ml IL-4 (StemCell Technologies) in 24-well plates for 3 days.

### Flow cytometry

The antibodies specific for FITC CD11c (clone N418), Fixable Viability Dye eFluor^®^ 780 and PE CD3 (clone 145-2C11) were purchased from eBioscience. The antibodies specific for PerCP MHCII (clone M5/114,15.2), Pacific Blue F4/80 (clone BM8), APC CD49b (clone DX5), FITC CD4 (clone RM4-5), Pacific Blue B220 (clone RA3-6B2) and PE/cy7 CD19 (clone 6D5) were purchased from Biolegend. V500 CD11b (clone M1/70), APC CD86 (clone GL1[RUO]), CD40 (clone 3/23) and CD24 (clone M1/69) were purchased from BD Bioscience. VioGreen CD8 (clone 53-6.7) was purchased from Miltenyi Biotec. Fluoresbrite plain YG 0.5 micron microspheres (2.5% solid-latex) were purchased from Polysciences, Inc. Flow cytometry was performed using Miltenyi Biotec MACSQuant analyzer and ImageStream^®^X Mark II Imaging Flow Cytometer. Cell sorting was accomplished with BD LSR II-B. Analysis was done using FlowJo software (FlowJo LLC).

### Total mRNA sequencing

Sequencing of cDNA was performed by the Illumina NextSeq 500 Sequencing System. RNA-Seq paired-end reads were aligned to the Ref Seq Release 77 mouse transcriptome reference using HISAT2 [30]. IsoEM2, an expectation–maximization algorithm for inference of isoform- and gene-specific expression levels from RNA-Seq data, was used to estimate gene expression levels [31]. Gene expression was reported as transcripts per million (TPM) units. Each gene was assigned the value of log_2_ (TPM + 1) to generate heat maps. IsoDE2 method was run for gene differential expression. Differential expressed genes (DEGs) were investigated by the ingenuity pathway analysis (IPA) software program, which can analyze the gene expression patterns using a scientific literature-based database (http://www.ingenuity.com). The Web-based tool Morpheus (https://software.broadinstitute. org/morpheus/) was used to generate heat maps of genes with assigned value of log2 (TPM + 1).

### Statistical analysis

P values for TCI scores comparisons were calculated using a two-tailed *t* test, using GraphPad Prism 5.0 (GraphPad). *P* < 0.05 was considered statistically significant.

## Results

### Dendritic cells but not macrophages mediate potent neoepitope-elicited tumor protection

**Fig. 1:**
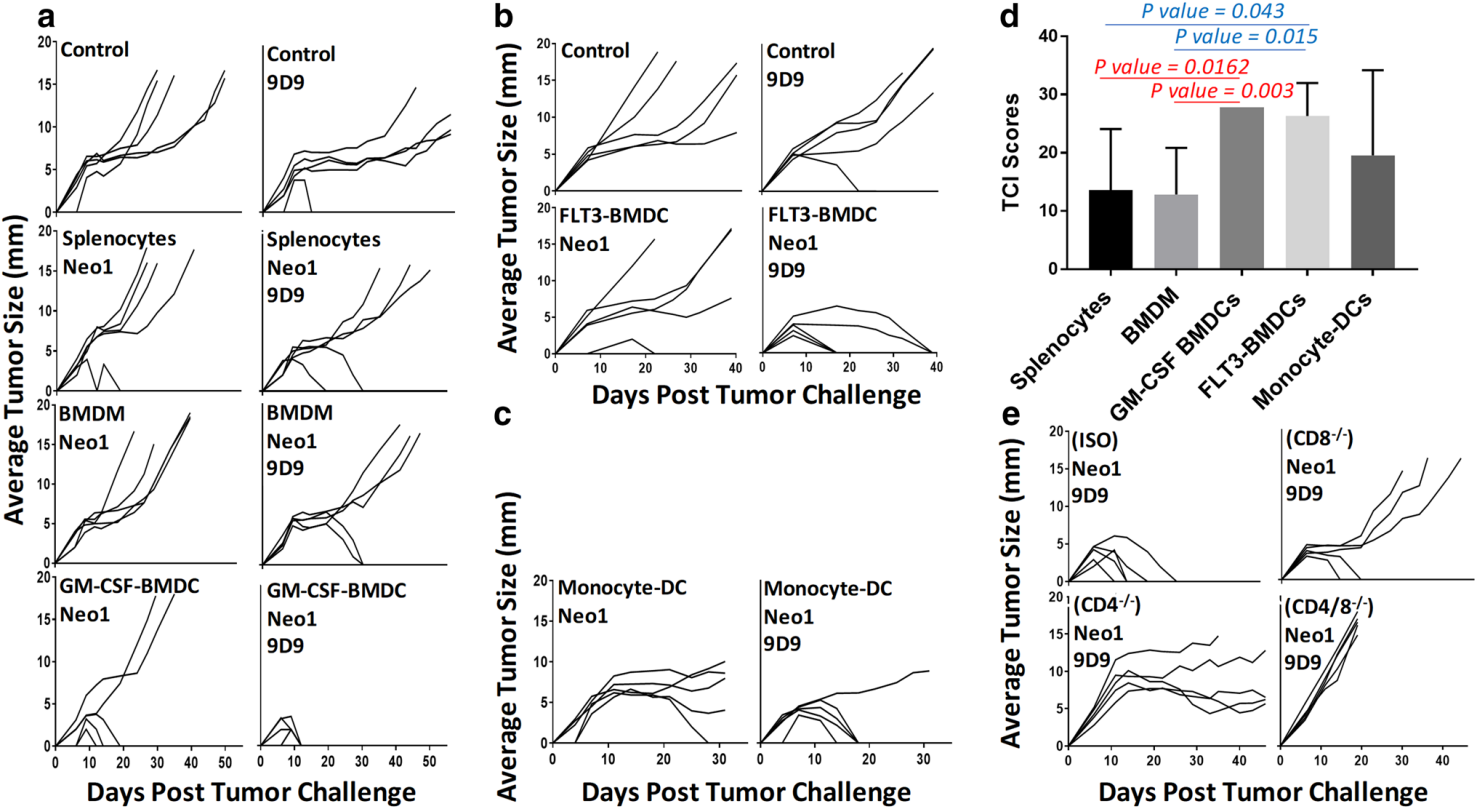
BMDCs are the most potent adjuvants. For all tumor growth graphs, each line represents tumor growth in a single mouse (*n* = 5 per group). **a** BALB/cJ mice were immunized twice, 1 week apart, with 100 µM Neo1-pulsed splenocytes (1.5 × 10^7^), GM-CSF-BMDCs (3 × 10^6^) or BMDM (3 × 10^6^); mice were administered 75 µg 9D9 antibody as indicated in “Materials and methods”. 7 days after the second immunization, mice were challenged with 95,000 Meth A cells and tumor growth was measured. **b, c** BALB/cJ mice were immunized with 100 µM Neo1-pulsed 3 × 10^6^ FLT3L-BMDCs (**b**) or Mo-DCs (**c**). Other details were the same as in **a. d** Total TCI scores for **a, b** and **c** data sets with 9D9 are shown. TCI scores of GM-CSF- and FLT3-BMDCs were statistically higher than BMDM and splenocytes (GM-CSF-BMDCs/BMDM *P* = 0.003, GM-CSF-BMDC/splenocytes *P* = 0.0162, FLT3-BMDCs/BMDM *P* = 0.015 and FLT3-BMDCs/splenocytes *P* = 0.043). **e** BALB/c mice were injected with 250 µg of CD8 or CD4 depletion antibodies (or isotype controls) as described in “Materials and methods”. Tumor challenge was the same as **a**. Experiments in various panels were repeated 2–10 times, with the exception of panel **c**, which was done only once. This experiment was not repeated since obtaining enough blood to isolate monocytes required over 60 mice per group

We used the mutant neoepitope Neo1 of the Meth A fibrosarcoma of BALB/cJ mice as the antigen for immunization. Mice were immunized twice, 1 week apart, with Neo1-pulsed splenocytes (1.5 × 10^7^), BMDM (3 × 10^6^), or GM-CSF-BMDCs (3 × 10^6^) as adjuvants. All mice were challenged with 9.5 × 10^4^ Meth A tumor cells subcutaneously, and tumor growth was measured twice a week (Fig. 1a, left panels). The DC-Neo1 immunized mice were the only group which showed tumor protection (with 3 of 5 mice displaying complete protection). Conversely, Neo1-pulsed macrophages immunization did not elicit any antitumor activity. Splenocytes and BMDM were phenotypically characterized using antibody markers for macrophages, DCs, B cells, CD4 and CD8 T cells (Supplementary Fig. 1).

The same immunization was attempted in the presence of anti-CTLA4 antibody (9D9 IgG2b) (Fig. 1a, right panels). The isotype control antibody (mouse IgG2b isotype control) did not have any effect on tumor rejection in over 10 experiments. The best tumor rejection (100%) was observed in mice immunized with BMDCs. We quantified the tumor rejection capacity of different immunization methods and statistically compared them using Tumor Control Index (TCI) scores [32]. The TCI score parameterizes and combines the tumor inhibition, tumor rejection, as well as tumor stability scores to yield a total TCI score, which numeral reflects the inhibition of the tumor growth in each group. TCI score of GM-CSF-BMDC group was significantly higher than that of macrophages (*P* = 0.003) and splenocytes (*P* = 0.0162) immunized groups (Fig. 1d).

Other types of DCs were also tested as adjuvants in the same setting of tumor rejection as in Fig. 1a. Mice were immunized with FLT3L-BMDCs, with or without Neo1 peptide and with or without 9D9 IgG2b (Fig. 1b). Complete (100%) rejection was observed in mice immunized with Neo1-pulsed FLT3L-BMDCs in the presence of 9D9 IgG2b (Fig. 1b). TCI score for the FLT3L-BMDC group was significantly higher than that of the macrophage (*P* = 0.015) and splenocyte (*P* = 0.043) groups (Fig. 1d). In order to test the adjuvanticity of Mo-DCs, mouse blood monocytes were harvested and differentiated into dendritic cells with GM-CSF and IL-4 and subsequently used for immunization. Four out of five Meth A tumors were rejected in the mice immunized with Mo-DCs (Fig. 1c). The adjuvanticities of GM-CSF-BMDC, FLT3L-BMDC and Mo-DC were statistically indistinguishable from each other (Fig. 1d); however, we consider GM-CSF BMDC to be the better adjuvants for two reasons: (1) the kinetics of tumor rejection seen in BMDC-immunized mice is clearly very different from that seen with Mo-DCs and FLT3L-DC. Please note Fig. 1a–c. All mice reached complete rejection in the case of BMDC-immunized mice within 10–12 days post-challenge. In Mo-DCs and FLT3L-DC-immunized mice however, tumor rejection occurred over 17–40 days (FLT3-DC), or 17–20 days (Mo-DCs). (2) There is almost no variability in tumor rejection in BMDC-immunized mice as evidenced by the numerous times this condition was repeated in subsequent figures.

CD8 and CD4-dependence of immunity elicited by Neo1-pulsed BMDC and 9D9 IgG2b immunization was tested by depleting mice of the respective cells as described in “Methods”. Tumor protection was observed to be CD8, as well as CD4 dependent (Fig. 1e).

### Adjuvanticity of GM-CSF-BMDCs derives from their role as antigen donor cells as well as antigen presenting cells

**Fig. 2:**
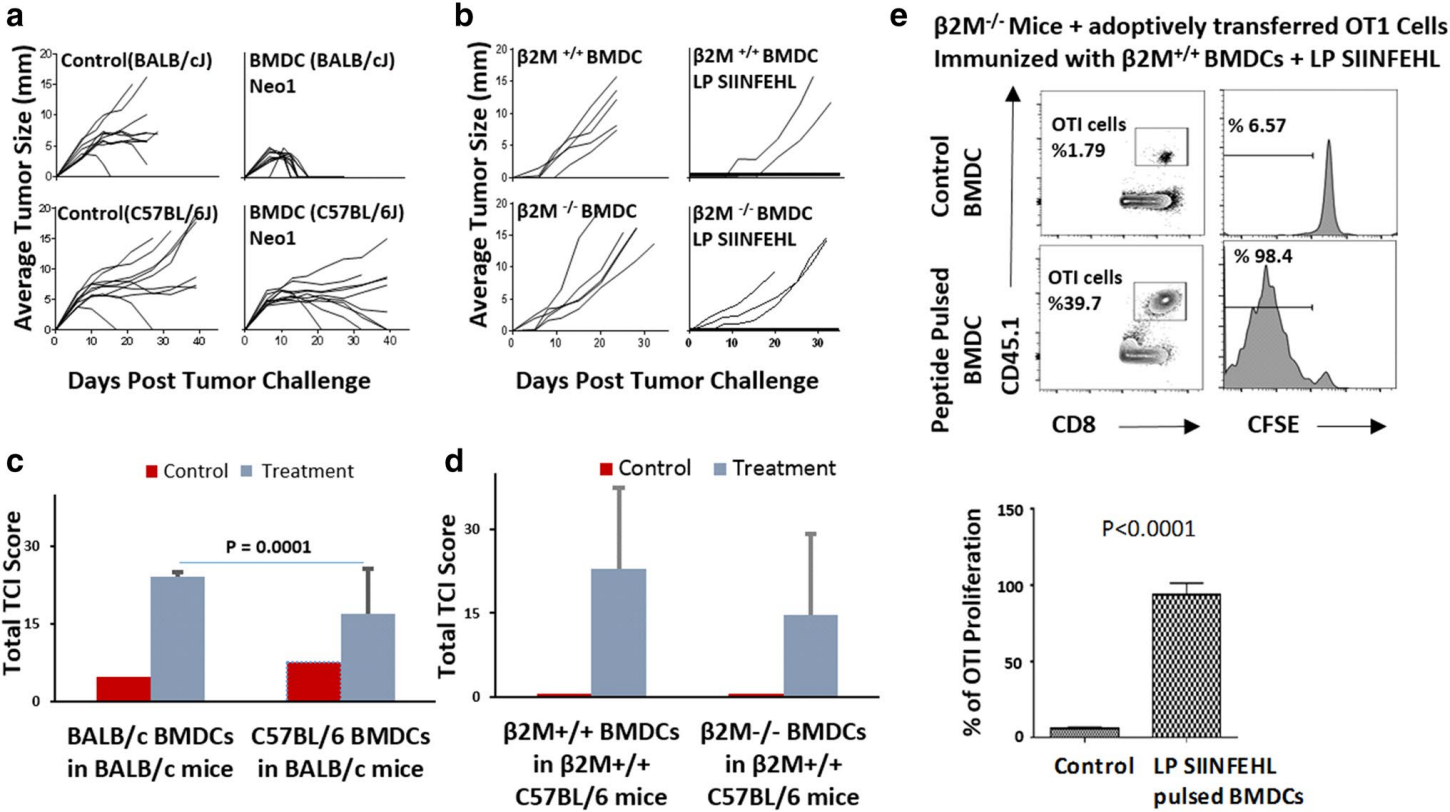
BMDCs act as ADCs as well as APCs. **a** BALB/cJ mice were immunized with Neo1-pulsed BALB/cJ or C57BL/6 BMDCs followed by 9D9 treatment, and tumor challenge as in Fig. 1. Tumor growth was measured (*n* = 10 per group). **b** C57BL/6 mice were immunized with LP SIINFEHL-pulsed β2M^−/−^ C57BL/6J BMDCs. 7 days after the second immunization, mice were challenged with 150,000 B16-OVA F0 tumor cells. Tumor growth was measured (*n* = 5 per group). **c** and **d** represent normalized TCI score for Fig. 2a, b. The average TCI score of BALB/cJ mice immunized with BALB/cJ BMDCs was significantly higher (*P* = 0.0001) than BALB/cJ mice immunized with C57BL/6 BMDCs. **e** CFSE-labeled OT-I CD8 + T cells were adoptively transferred into two groups (*n* = 3 per group) of β2M^−/−^ mice, on day − 3. After 24 h, all mice were immunized intradermally with BMDCs alone or LP SIINFEHL-pulsed BMDCs. Draining lymph nodes were harvested from individual mice 72 h after OT-I transfer and dilution of CFSE on CD45.1+ gated OT-I CD8+ T cells was analyzed. The percentage of OT-I CD8 T-cell proliferation in β2M^−/−^ mice immunized with β2M^+/+^ BMDCs was significantly higher (*P* < 0.0001) than in the control group. Experiments in panels **a**–**d** were done in whole or in parts, at least twice

In order to dissect the role of BMDCs as ADCs versus APCs, BMDCs of two different haplotypes were used as adjuvant. BALB/cJ mice were immunized with BMDCs derived from BALB/cJ (H-2^d^) or C57BL/6 (H-2^b^) mice, with or without Neo1 peptide and with 9D9 IgG2b (Fig. 2a). The mice immunized with BALB/cJ-derived BMDCs, Neo1, in the presence of 9D9 IgG2b rejected the Meth A tumor challenge completely (100%) and rapidly (< 20 days). For mice immunized with C57BL/6J BMDCs, the Neo1 group also rejected the Meth A tumors, albeit less completely (50%) and over a longer time course (between 20 and > 40 days). Normalized TCI score of mice immunized with Neo1-pulsed isogeneic BMDCs is significantly higher (*P* = 0.0001) than in mice immunized with Neo1-pulsed allogeneic BMDCs (Fig. 2c). The higher tumor protection in the mice immunized with Neo1-pulsed (syngeneic) BALB/cJ BMDCs might be due to the APC function of the syngeneic BMDCs (i.e., BMDCs with self MHC might serve both as antigen reservoirs as well as APCs).

In order to further dissect the difference between ADC and APC roles of BMDCs, we used MHC I-expressing and non-expressing DCs from β2 microglobulin^+/+^ (β2M^+/+^) or β2M^−/−^ mice. β2M^−/−^ are available only in the C57BL/6 and not the BALB/c background where Neo1 may be used. For this specific purpose, we switched to the use of the chicken ovalbumin (and its well-known, dominant K^b^-restricted epitope SIINFEKL) for this experiment only. C57BL/6 mice were immunized with LP SIINFEHL (18-mer) pulsed β2M^+/+^ or β2M^−/−^ C57BL/6 BMDCs twice, 1 week apart. (β2M^−/−^ DCs can act only as ADCs and not as APCs.). 1 week after the second immunization, mice were challenged with 1.5 × 10^5^ B16-OVA tumor cells and tumor growth was measured (Fig. 2b). Mice immunized with normal BMDCs (β2M^+/+^) showed tumor protection (60% complete protection) while mice immunized with β2M^−/−^ BMDCs showed less robust tumor protection (40% complete protection). Normalized TCI score of the mice immunized with Neo1-pulsed β2M^+/+^ BMDCs was higher than the mice immunized with Neo1-pulsed β2M^−/−^ BMDCs (Fig. 2d), although the difference was not statistically significant in and of itself. However, a highly significant difference (*P* = 0.0001) between syngeneic and allogeneic BMDCs was seen in the tumor rejection data (Fig. 2a, c).

A more stringent and quantitative test for the role of BMDCs as APCs was devised. OVA-specific CD8^+^ T cells were enriched from single cell suspension of spleen, mesenteric lymph node (LN), and skin draining LNs of CD45.1 OT-I mice. OT-I CD8^+^ T cells were then labeled with CFSE and the labeled OT-I CD8^+^ T cells were adoptively transferred into two groups of β2M^−/−^ mice (β2M^−/−^ mice have no competent MHCI APCs). Mice were immunized with LP SIINFEHL-pulsed β2M^+/+^ BMDCs. Draining LNs were harvested from individual mice, and dilution of CFSE in gated CD45.1^+^ OT-I CD8^+^ T cells was analyzed (Fig. 2e). β2M^−/−^ mice immunized with β2M ^+/+^ DCs supported vigorous proliferation of OTI cells (*P* < 0.0001) indicating that the immunizing BMDCs acted as APCs in β2M^−/−^ mice (Fig. 2e bottom panel). β2M^+/+^ mice immunized with OVA in any form always support vigorous proliferation of OTI cells.

### CD11c^+^ MHCII^lo^ GM-CSF-BMDCs mediate the most potent neoepitope-elicited tumor protection

**Fig. 3:**
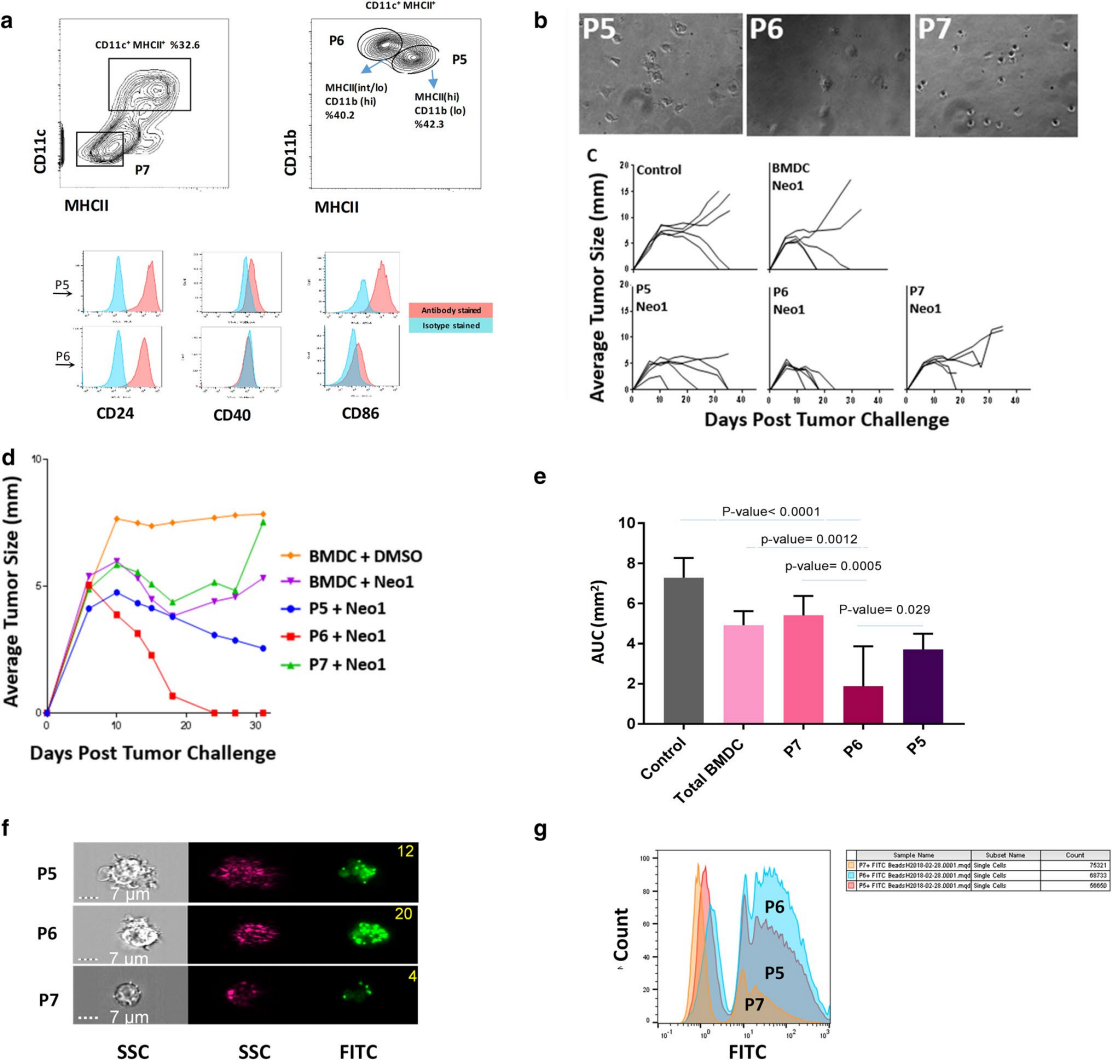
Sub-populations of BMDCs have distinct tumor rejection capacities. **a** The phenotype of GM-CSF-BMDCs cultures at day 7. CD11c^+^MHCII^+^ BMDCs (**a**, left) are divided further based on the CD11b expression (**a**, right; P6: MHCII^lo^ CD11b^hi^ and P5: MHCII^hi^ CD11b^lo/int^). Boxes represent gates and percentage of cells in each gate. Histograms indicate surface expression of the indicated markers by P5 (red) and P6 (blue) sub-sets. **b** shows the photomicrograph of P5, P6 and P7 cell sub-populations (200×). **c** shows tumor growth in BALB/cJ mice immunized with Neo1-pulsed 500,000 un-fractionated BMDCs, P5, P6 or P7 cells. All the immunizations were performed twice, 1 week apart with 9D9 treatment. Mice were challenged with Meth A cells and tumor growth monitored as in Fig. 1 (*n* = 5 per group). **d** shows the group average of tumor growth for panel **c. e** shows area under the curve (AUC) scores for each group of panel **c**. The AUC was calculated from day 6–31 since all groups showed uniform growth from day 0–6. The AUC value for P6 population was significantly lower than that of P5 (*P* = 0.029), P7 (*P* = 0.0005), total BMDC (*P* = 0.0012) and the control group (*P* < 0.0001). **f** Photomicrographs of P5, P6 and P7 sub-populations that were incubated with FITC microbeads. The right and middle panels represent the side-scattered images of the cells in the bright and dark field, respectively. The right panel shows the FITC channel. The number of the beads taken up by each sub-population is indicated at the top right corner of the image. **g** Cells were incubated with FITC and acquired by MACS Quant machine. *X* axis and *Y* axis represent the FITC channel and count, respectively. Experiments in panels **a**–**g** were done at least two times

GM-CSF-BMDCs were sorted based on expression of CD11c and MHC class II into three sub-populations similar to the sorting strategy adopted earlier [33] (Fig. 3a): undifferentiated cells without CD11c and MHCII expression (P7), CD11c^+^ MCHII^lo^ cells (P6) and CD11c^+^ MHCII^hi^ cells (P5). P5 and P6 sub-populations were also characterized for the expression of CD24, CD40 and CD86 co-stimulatory molecules as well CD11b (Fig. 3a, bottom panels). P5, P6 and P7 cells were also analyzed via light microscopy (Fig. 3b). Between P5 and P6 sub-populations, P5 showed higher expression of co-stimulatory molecules, lower expression of CD11b and a larger number of dendrites per cell. Hence, P5 resembled mature DCs and P6 appeared to have characteristics of immature DCs [34]. This conclusion is also consistent with the expression of various surface markers as deduced by RNA sequencing analysis that are used to characterize immature and mature DCs. Table 1 shows that the P5 sub-population displayed higher expression of CD40, CD24, CD80/86 and MHCII as compared to the P6 sub-population (Table 1).

**Table 1:** Expression of transcripts for selected surface markers on P5 and P6 cell sub-populations

Surface markers	Genes	P5 (TPM^a^)	P6 (TPM^a^)	Log_2-_fold change (P6/P5)^b^
CD209a	Cd209a	146	6	− 4.6
CD24a	Cd24a	549	323	− 0.8
CD80	Cd80	32	16	− 0.9
CD40	Cd40	10	2	− 2.5
CD86	Cd86	109	16	− 2.7
Histocompatibility 2, class II antigen A, beta 1	H2-Ab1	3738	325	− 3.5
Histocompatibility 2, class II antigen A, alpha	H2-Aa	5454	393	− 3.8
Histocompatibility 2, M region locus 2	H2-M2	3	0	− 3.8

^a^Transcripts per million

^b^The Log2-fold change of P5 /P6 was computed using IsoDE2 tool with a statistical significance level of 0.05 (https://toolshed.g2.bx.psu.edu/view/saharlcc/isoem2_isode2/)

Mice were immunized with Neo1 pulsed P5, P6, P7 or whole BMDCs as control (5 × 10^5^/mouse) in the presence of 9D9 and challenged as in Fig. 1. A lower dose of BMDCs (5 × 10^5^/mouse) was used deliberately, so as to be able to see the activity in a titratable range; indeed, at this dose of total BMDCs, significant but less robust rejection of tumors was seen, compared with that observed in Figs. 1 and 2 where a higher dose of BMDCs (3 × 10^6^/mouse) was used (Fig. 3c). The P7 sub-population showed no adjuvanticity (*P* = 0.593). However, the highest and highly significant adjuvanticity was observed in the mice immunized with the P6 sub-population where all mice (5/5) showed complete tumor regression with a rapid kinetics (P5 compared with P6, *P* = 0.029). Data with individual mice are shown in Fig. 3c and pooled data from each group in Fig. 3d. Area under the curve values for the mice immunized with the P6 sub-population is significantly lower than the corresponding values in mice immunized with P5 and P7 sub-populations (Fig. 3e). Hence, P6 yielded better tumor rejection than P5 and P7 sub-populations. The sorted P5, P6 and P7 sub-populations were incubated with 0.5-µm FITC microspheres for 30 min to test the capacity of antigen uptake of each sub-population. Cells were thoroughly washed to remove excess beads from the cell surface. Using ImageStreamX Mark II Imaging Flow Cytometer (Fig. 3f) and MACS Quant (Fig. 3g) the number of beads taken up by each sub-population was quantified. The highest number of internalized beads was observed in the P6 sub-population. Around 71%, 25% and 32% (of P7, P6 and P5 cells, respectively) were observed to not have taken up any beads. The group that was able to uptake the highest percentage of more than 3 beads was P6 (28.9%) while the P5 and P7 percentages were 21.7 and 2.51%, respectively (Supplementary Fig. 2).

**Fig. 4:**
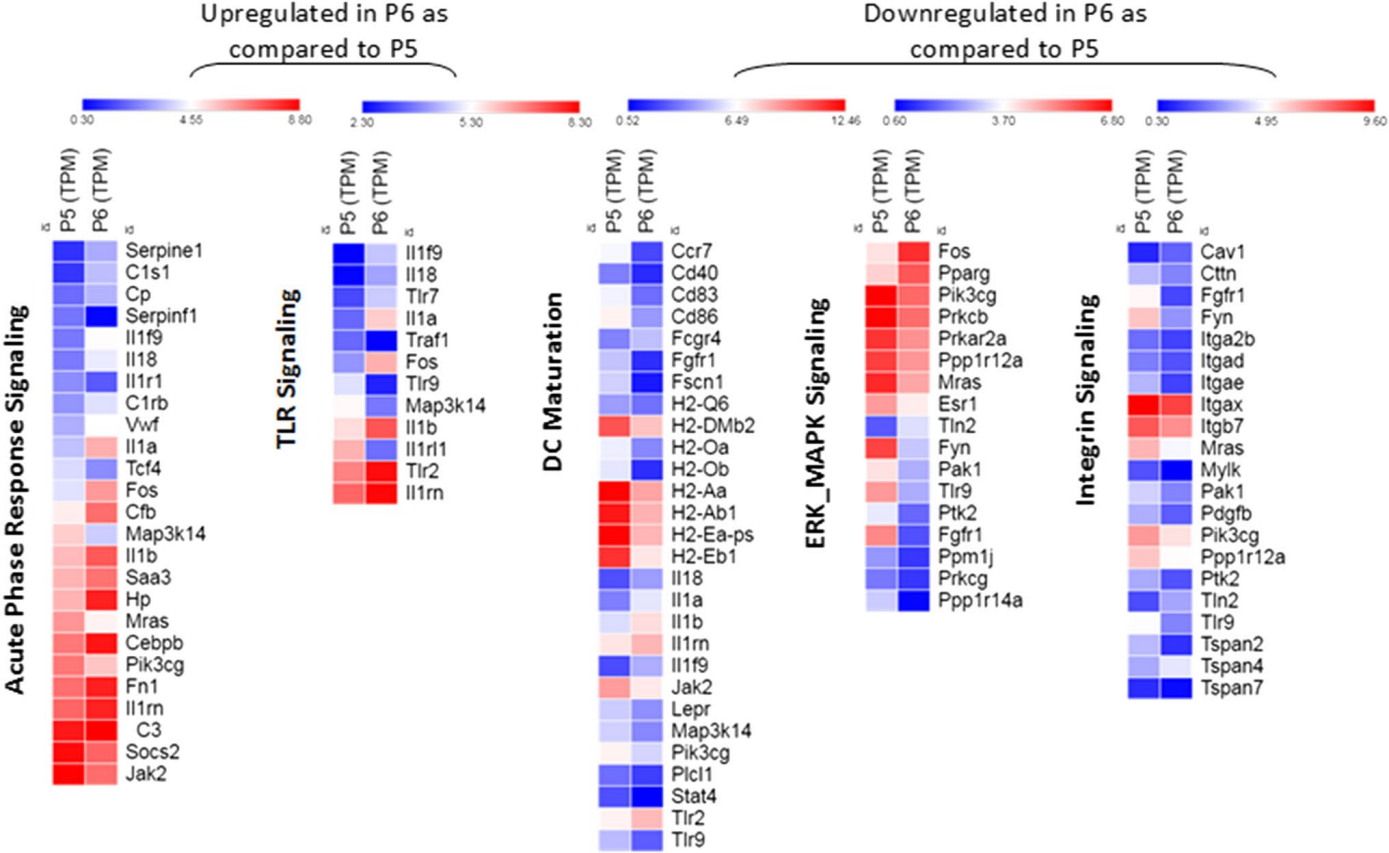
Heat map of the significant pathways selected for the P5 and P6 cell sub-populations using the IPA tools. IPA identified 869 up- and down-regulated genes in P6 compared to P5, eligible for pathways analysis. Canonical pathways were identified and analyzed from the IPA libraries. *P* values < 0.05 were used to define differentially expressed genes. This experiment has been done once

Using total mRNA sequencing, differential gene expression analysis was performed on the P5 and P6 sub-populations. Both sub-populations showed RNA expression signatures for DCs as well as macrophages, although the P5 sub-population showed higher expression level of all markers tested. P5 and P6 sub-populations were compared for maturation phenotype using RNA-Seq (Table 1). There are other significant transcriptional differences between the P5 and P6 sub-populations as well (Table 2). The expression of CD91 and LOX1, both heat shock protein receptors as well as two mannose receptors and selected toll-like receptors (TLR1, TLR2, and TLR6) were increased in P6 as compared to P5. The increase was more substantial for some genes (LOX1, CD91 and TLR2) than for others. CD36 (scavenger receptor) was the only major receptor that showed substantial reduction in P6 as compare to P5 (> four-fold). The heat maps of the transcriptional data (Fig. 4) show that the P5 sub-population expressed a higher level of genes involved in DC maturation, migration (integrin signaling) and proliferation (ERK/MAPK signaling) while pathways involved in TLR signaling and acute-phase response signaling predominated in the P6 sub-population. The individual genes of each pathway that show the most difference between the P5 and P6 sub-populations are shown in Supplementary Tables 1 and 2.

**Table 2:** Transcriptional profile of selected receptors in P5 and P6 cell sub-populations

Protein name	Genes	P5 (TPM^a^)	P6 (TPM^a^)	Log_2_ fold change (P6/P5)^b^
CD91	Lrp1	32.40	66.64	1.04
LOX-1	Olr1	4.38	21.75	2.31
Mannose receptor C-type 1	Mrc1	184.00	319.81	0.79
Macrophage scavenger receptor 1	Msr1	221.37	347.86	0.65
TLR1	Tlr1	4.55	8.88	0.94
TLR2	Tlr2	99.55	260.73	1.38
TLR6	Tlr6	14.08	22.81	0.68

^a^Transcripts per million

^b^The Log2 fold change of P5 /P6 was computed using IsoDE2 tool with a statistical significance level of 0.05 (https://toolshed.g2.bx.psu.edu/view/saharlcc/isoem2_isode2/)

## Discussion

Here, using a bonafide neoepitope tumor rejection antigen, we show that macrophages are not effective adjuvants, but DCs are. GM-CSF-BMDCs, FLT3L-BMDCs, and Mo-DCs are all excellent adjuvants, although the GM-CSF DCs seem to be the more effective. GM-CSF-BMDCs have been previously characterized as a heterogeneous population consisting of un-differentiated cells, DC-like cells as well as macrophage-like cells [33]. Here, we observe that this heterogeneous population consists of cells with cell surface markers of DCs as well as macrophage without a clear demarcation between DC-like and macrophage-like cells; instead the heterogeneity observed by us is in the maturation status of these DCs. One major sub-population, P5, is more akin to mature DCs, while the P6 sub-population is similar to immature DCs. It is possible that differences in culture conditions (namely, GM-CSF alone in our study as compared with GM-CSF/IL-4 in the study of Helft et al.) are responsible for the differences. Most interestingly, we observe that while both P5 and P6 sub-populations are effective adjuvants, the P6 sub-population is clearly more effective than the P5. The immature DC phenotype of the P6 sub-population, with a higher capacity for antigen uptake, and possibly a higher antigen sequestering capacity, may be responsible for this superior activity. Luketic et al. [35] and Li et al. [36] have previously demonstrated that DCs have a unique ability to sequester antigenic epitopes or their precursors for extended periods of time, up to several weeks. Here, we speculate that the P6 sub-population has a better antigenic sequestering ability than the P5. In terms of transcriptional profiles as well, the P6 sub-population expresses higher levels of a variety of immunologically important receptors including heat shock protein receptors CD91 and LOX1, mannose receptors as well as select TLRs.

Finally, our studies resolve the question of mechanisms by which exogenous DCs mediate CD8 immunity. Previous studies have argued that DCs-as-adjuvants act as ADCs only [23], or APCs only [22]. Using two distinct methods of analysis, our results show clearly that DCs act in both capacities, although un-equally. The major contribution towards the adjuvanticity of DCs derives from their role as ADCs, possibly because of the unusual capacity of DCs to sequester antigen for prolonged periods of time [36]. The identification of a specific sub-set of DCs with the highest adjuvanticity as well as a better understanding of the mechanisms of their adjuvanticity lays a foundation for further investigations and comparison of CD11c^+^ MCHII^lo^ cell sub-population to a possible human DC counterpart in order to use DCs as highly effective adjuvants in neoepitope-based cancer vaccines.

### Electronic supplementary material

Below is the link to the electronic supplementary material.

Supplementary material 1 (PDF 399 KB)

## Abbreviations

ADC: Antigen donor cell
APC: Antigen presenting cell
BMDC: Bone marrow-derived dendritic cell
BMDM: Bone marrow-derived macrophage
DC: Dendritic cell
DEG: Differential expressed gene
FLT3L: FMS-like tyrosine kinase 3 ligand
GM-CSF: Granulocyte-macrophage colony-stimulating factor
IPA: Ingenuity pathway analysis
LN: Lymph node
LP: Long peptide
M-CSF: Macrophage colony-stimulating factor
Mo-DCs: Monocyte-derived DCs
PBMC: Peripheral blood mononuclear cell
TLR: Toll like receptors
β2M: β2 microglobulin
