# Supplementary material for: CD11c+ MHCIIlo GM-CSF-bone marrow-derived dendritic cells act as antigen donor cells and as antigen presenting cells in neoepitope-elicited tumor immunity against a mouse fibrosarcoma
Source: Cancer Immunol Immunother. 2018 Jul 20;67(9):1449–59. doi: 10.1007/s00262-018-2202-4 (PMC6132860; doi:10.1007/s00262-018-2202-4)
Supplement: Supplementary file 1 — Supplementary material 1 (PDF 399 KB) [file 262_2018_2202_MOESM1_ESM.pdf]

Cancer Immunology, Immunotherapy (submitted in 2018)- Hakimeh Ebrahimi-Nik et. al

Supplementary Table 1. Pathways that are significantly upregulated in P6 compared to P5 sub-populations by IPA

Pathways	Genes	Log_2 Fold Change (P6/P5)	P5 (TPM[a])	P6 (TPM[a])
Acute Phase Response Signaling (25/158 genes)	C3	1.1	335.9	747
	C1rb	1.4	5.9	15.4
	C1s1	2.9	1.3	10
	Cebpb	1.7	109	349.8
	Cfb	2.1	27.7	123.8
	Cp	1.4	3.3	8.7
	Fn1	1.3	123.3	307.4
	Fos	2.2	15.6	74.3
	Hp	2.5	54.9	304.4
	Il18	2.1	4.1	17.6
	Il1a	2.5	10.5	58.3
	Il1b	1.6	51.2	155.9
	Il1r1	-1.1	5.3	2.5
	Il1rn	1.1	136	298.8
	Il1f9	2.5	4	23.3
	Jak2	-1.8	439.4	125.6
	Map3k14	-1.7	40.5	12.1
	Mras	-1.6	78.1	26.2
	Pik3cg	-1.3	110.3	45
	Saa3	1.1	54.6	116.8
	Serpine1	2.6	1.2	7.9
	Serpinf1	-3.7	3.8	0.3
	Socs2	-1.5	390.7	139.3
	Tcf4	-1.4	14.2	5.3
	Vwf	1.4	8.2	22.4
TLR Signaling (12/72 genes)	Fos	2.2	15.6	74.3
	Il18	2.1	4.1	17.6
	Il1a	2.5	10.5	58.3
	Il1b	1.6	51.2	155.9
	Il1rl1	-2.7	73.1	11.1
	Il1rn	1.1	136	298.8
	Il1f9	2.5	4	23.3
	Map3k14	-1.7	40.5	12.1
	Tlr2	1.4	107.6	282.3
	Tlr7	1.6	7.9	24.8
	Tlr9	-2.4	29.7	5.5
	Traf1	-1.4	10.6	4

[a] Transcripts per Million

Supplementary Table 2. Pathways that are significantly downregulated in P6 compared to P5 sub-populations by IPA

Pathways	Genes	Log_2 Fold Change (P6/P5)	P5 (TPM[a])	P6 (TPM[a])
**Dendritic Cell Maturation (28/168 genes)**	**Ccr7**	**-4.5**	**79.1**	**3.5**
	**Cd40**	**-2.5**	**10.4**	**1.8**
	**Cd83**	**-3.2**	**74.2**	**7.8**
	**Cd86**	**-2.7**	**109.3**	**16.4**
	**Fcgr4**	**1.5**	**11.1**	**31.7**
	**Fgfr1**	**-4.1**	**34.2**	**2**
	**Fscn1**	**-5.1**	**40.1**	**1.2**
	**H2-Q6**	**-1.1**	**17.2**	**8**
	**H2-DMb2**	**-2.6**	**1495**	**242.9**
	**H2-Oa**	**-2.5**	**65.7**	**11.6**
	**H2-Ob**	**-4.8**	**60.4**	**2.1**
	**H2-Aa**	**-3.8**	**5337.3**	**385.1**
	**H2-Ab1**	**-3.5**	**3715.8**	**323.6**
	**H2-Ea-ps**	**-4.1**	**5228.8**	**294.1**
	**H2-Eb1**	**-4.2**	**2511.4**	**135.4**
	**Il18**	**2.1**	**4.1**	**17.6**
	**Il1a**	**2.5**	**10.5**	**58.3**
	**Il1b**	**1.6**	**51.2**	**155.9**
	**Il1rn**	**1.1**	**136**	**298.8**
	**Il1f9**	**2.5**	**4**	**23.3**
	**Jak2**	**-1.8**	**439.4**	**125.6**
	**Lepr**	**-1.5**	**39.1**	**13.7**
	**Map3k14**	**-1.7**	**40.5**	**12.1**
	**Pik3cg**	**-1.3**	**110.3**	**45**
	**Plcl1**	**-1.2**	**7.3**	**3.1**
	**Stat4**	**-3.4**	**4.1**	**0.4**
	**Tlr2**	**1.4**	**107.6**	**282.3**
	**Tlr9**	**-2.4**	**29.7**	**5.5**
**Integrin Signaling (20/211 genes)**	**Cav1**	**1.6**	**1**	**3.2**
	**Cttn**	**-1.1**	**11.9**	**5.4**
	**Fgfr1**	**-4.1**	**34.2**	**2**
	**Fyn**	**-3.1**	**62.3**	**7.1**
	**Itga2b**	**-1.1**	**4**	**1.9**
	**Itgae**	**-2.6**	**11.1**	**1.8**
	**Itgax**	**-1.2**	**780.5**	**328.4**
	**Itgb7**	**-1.1**	**261.3**	**125.1**
	**Mras**	**-1.6**	**78.1**	**26.2**
	**Mylk**	**-3.6**	**2.6**	**0.2**
	**Pak1**	**-1.5**	**15.8**	**5.6**
	**Pdgfb**	**-1.7**	**10.3**	**3.1**
	**Pik3cg**	**-1.3**	**110.3**	**45**
	**Ppp1r12a**	**-1.1**	**64.6**	**30.4**
	**Ptk2**	**-1.9**	**9.9**	**2.6**
	**Tln2**	**2**	**2.2**	**8.8**
	**Tlr9**	**-2.4**	**29.7**	**5.5**
	**Tspan2**	**-3.1**	**11.8**	**1.3**
	**Tspan4**	**1.1**	**9.7**	**21.4**
	**Tspan7**	**-1.3**	**1.1**	**0.4**
**ERK/MAPK Signaling (16/191 genes)**	**Esr1**	**-1**	**29**	**14.2**
	**Fgfr1**	**-4.1**	**34.2**	**2**
	**Fos**	**2.2**	**15.6**	**74.3**
	**Fyn**	**-3.1**	**62.3**	**7.1**
	**Mras**	**-1.6**	**78.1**	**26.2**
	**Pak1**	**-1.5**	**15.8**	**5.6**
	**Pik3cg**	**-1.3**	**110.3**	**45**
	**Pparg**	**1.5**	**18.2**	**52.4**
	**Ppm1j**	**-1.6**	**4.4**	**1.4**
	**Ppp1r14a**	**-3.5**	**7.4**	**0.6**
	**Prkar2a**	**-1.2**	**71.1**	**31.8**
	**Prkcb**	**-1.3**	**107.7**	**43.6**
	**Prkcg**	**-1.1**	**3.2**	**1.4**
	**Ptk2**	**-1.9**	**9.9**	**2.6**
	**Tln2**	**2**	**2.2**	**8.8**
	**Tlr9**	**-2.4**	**29.7**	**5.5**

[a] Transcripts per Million

Supplementary Figure 1.

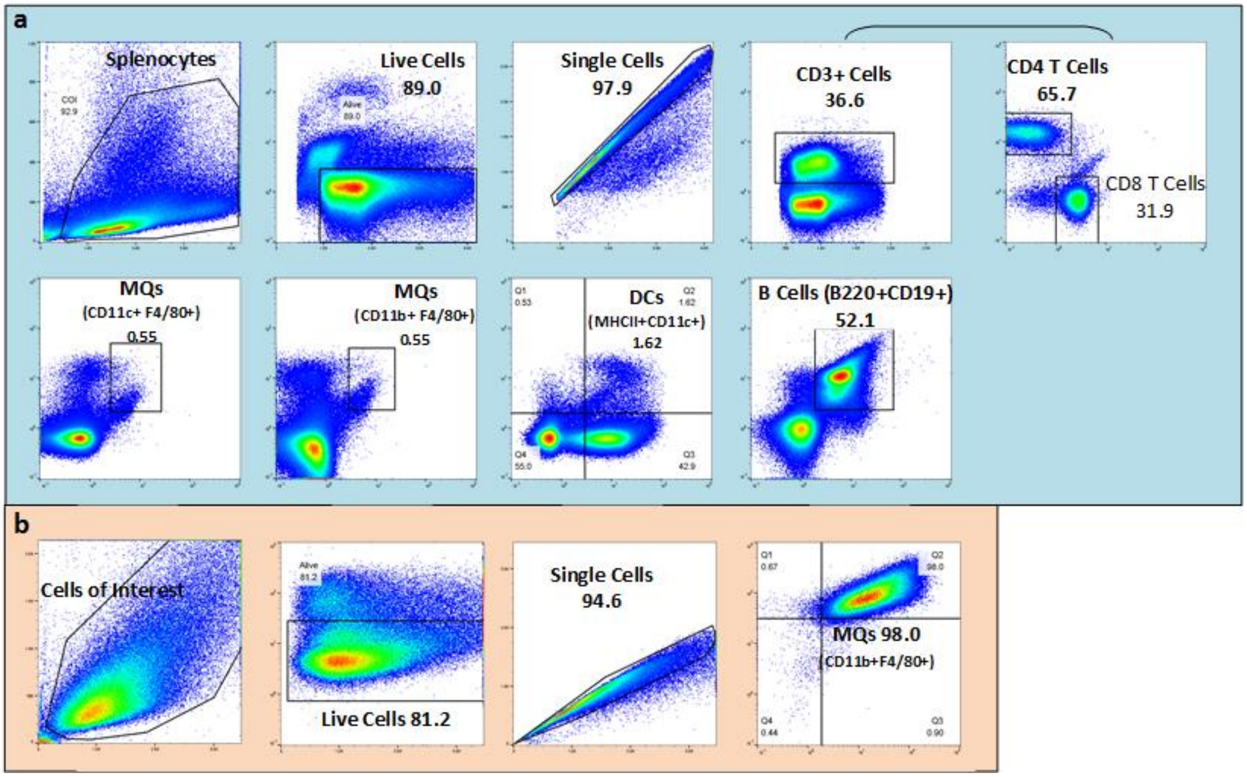

The phenotype of BALB/cJ splenocytes (a) and bone marrow derived macrophages (b). Boxes represents gates and percentage of cells in each gate.

Supplementary Figure 2.

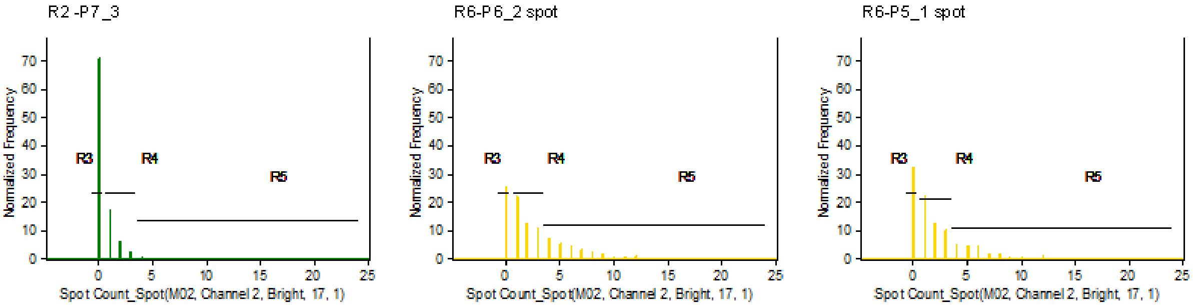

Spot Count_Spot(M02, Channel 2, Bright, 17, 1)

Population	Count	%Gated
R2 & R1	1037	100
R3 & R2 & R1	737	71.1
R4 & R2 & R1	274	26.4
R5 & R2 & R1	26	2.51

Spot Count_Spot(M02, Channel 2, Bright, 17, 1)

Population	Count	%Gated
R6	1023	100
R3 & R6	260	25.4
R4 & R6	467	45.7
R5 & R6	296	28.9

Spot Count_Spot(M02, Channel 2, Bright, 17, 1)

Population	Count	%Gated
R6	989	100
R3 & R6	322	32.6
R4 & R6	452	45.7
R5 & R6	215	21.7

Using ImageStreamX Mark II Imaging Flow Cytometer the number of the beads in each sub population were quantified. X-axis shows the number of beads and Y-axis indicates the percentage of the cells. R3 gate is representative of the cells that were not able to take up any beads. R4 shows the percentage of the cells that took up 1-3 beads and R5 represents the percentage of the cells that had more than 3 beads. The tables shows the statistics for each graph.
